# Sjögren Disease—B Cells at the Brink: From Autoimmunity to Lymphomagenesis and the Rise of Novel B Cell–Targeted Therapies

**DOI:** 10.1002/art.43404

**Published:** 2026-01-21

**Authors:** Rachael A. Gordon, Sara S. McCoy

**Affiliations:** ^1^ Department of Medicine University of Pittsburgh School of Medicine Pittsburgh Pennsylvania; ^2^ Department of Medicine University of Wisconsin School of Medicine and Public Health Madison; ^3^ Department of Medicine University of Wisconsin Carbone Comprehensive Cancer Center Madison

## Abstract

Sjögren disease (SjD) is a common systemic autoimmune disorder characterized by inflammation of the exocrine glands, resulting in dryness. Patients frequently exhibit extraglandular manifestations affecting various organ systems. To date, there are no US Food and Drug Administration (FDA)‐approved disease‐modifying therapies for SjD. In this review, we explore the expanding field of SjD endotyping as a tool to enhance patient stratification, prognostication, and clinical decision‐making. SjD endotypes driven by heightened B cell activity are linked to increased lymphoma risk. B cells play a central role in SjD pathogenesis by producing autoantibodies, presenting antigens, and releasing proinflammatory cytokines. These functions contribute not only to autoimmunity but also to lymphomatous transformation. We illustrate these concepts through the case of a patient with SjD who developed parotid mucosa‐associated lymphoid tissue lymphoma after years of recurrent glandular swelling—highlighting a common yet challenging scenario for practicing rheumatologists. Using this case as a framework, we examine the pathobiology of B cells in SjD that drive autoreactivity and lymphomagenesis. Finally, we review emerging B cell–targeted therapies that reflect a broader shift in the SjD treatment landscape from symptomatic management to targeted therapies grounded in disease immunopathology.

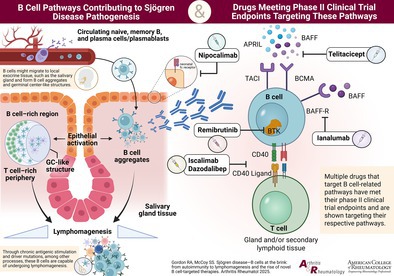

## Introduction

**Figure 1:**
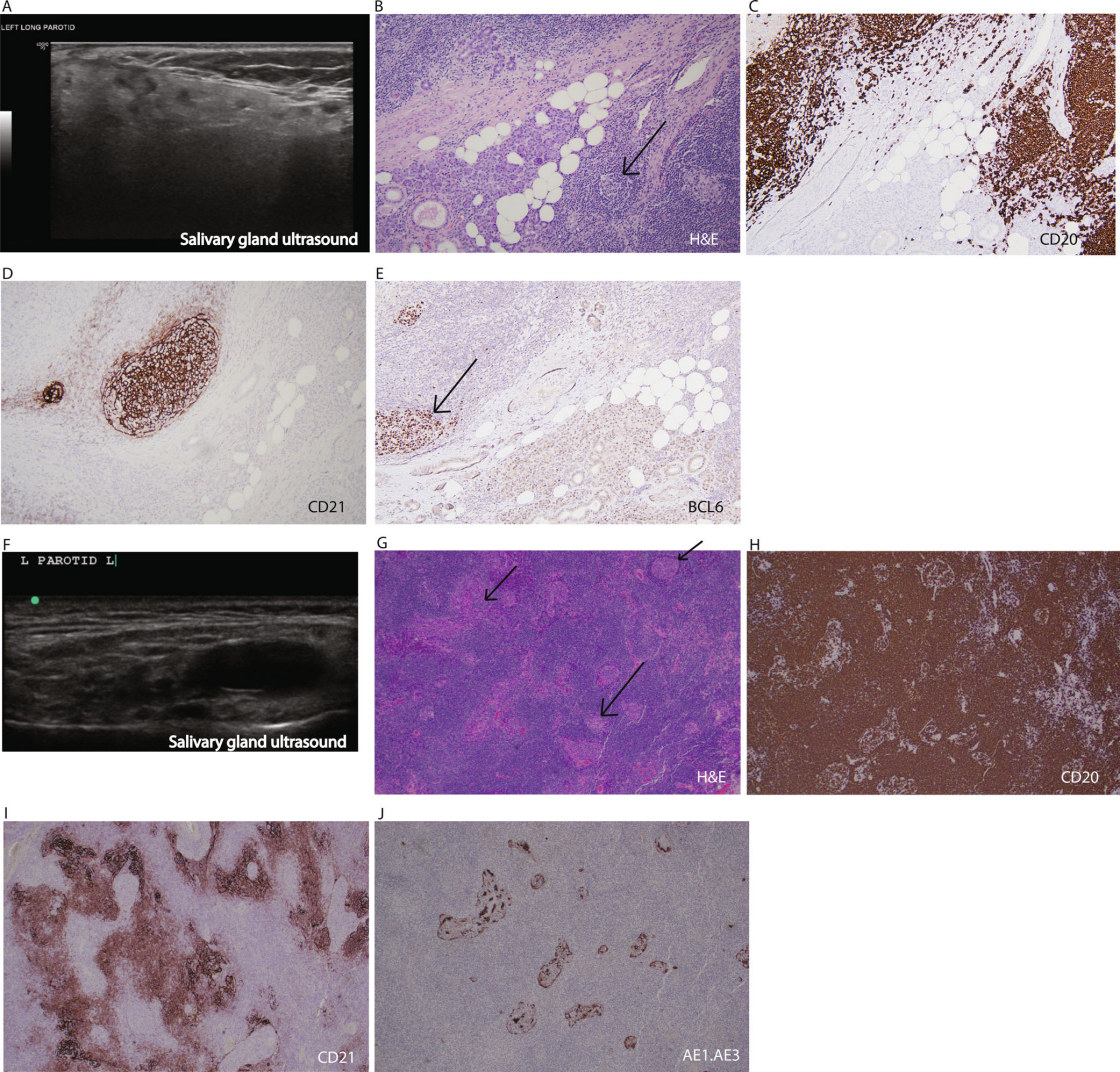
Early and late parotid pathology as case examples of lymphoma development. (A–D) Example of early imaging and pathology features in a case that would be considered at risk of progressing to lymphoma. (A) Salivary gland ultrasound shows scattered anechoic and hypoechoic changes involving <50% of the parotid tissue (view of left parotid gland, longitudinal). (B) Hematoxylin and eosin (H&E) shows lymphoepithelial sialadenitis (black arrow). (C‐E) Staining is notable for B cell predominant infiltrate (CD20) and the presence of germinal centers (CD21 & BCL6 [black arrow]). (F–J) Example of progression to lymphoma with imaging and pathology features. (F) Salivary gland ultrasound shows a dominant anechoic lesion in the setting of diffusely abnormal salivary gland tissue. (G) H&E shows atypical lymphoid infiltrate, consisting of small‐ to intermediate‐sized variably monocytoid‐appearing lymphocytes, proliferating in a nodular manner with frequent lymphoepithelial lesions observed. There are occasional small, irregular residual fragments of germinal centers and no morphologic features of large cell transformation (black arrows). (H‐J) CD20 staining shows predominance of B cells with coexpression of BCL2 and are associated with irregularly shaped, somewhat serpiginous CD21‐positive follicular dendritic cell meshworks. The neoplastic lymphocytes are negative for CD5, CD10, BCL6, and cyclin D1. The few small, residual germinal centers are negative for BCL2. (I) AE1.AE3 highlights epithelial cells within lymphoepithelial lesions. Credit goes to pathologists Drs Wei Huang and Erica Reinig.

**Figure 2:**
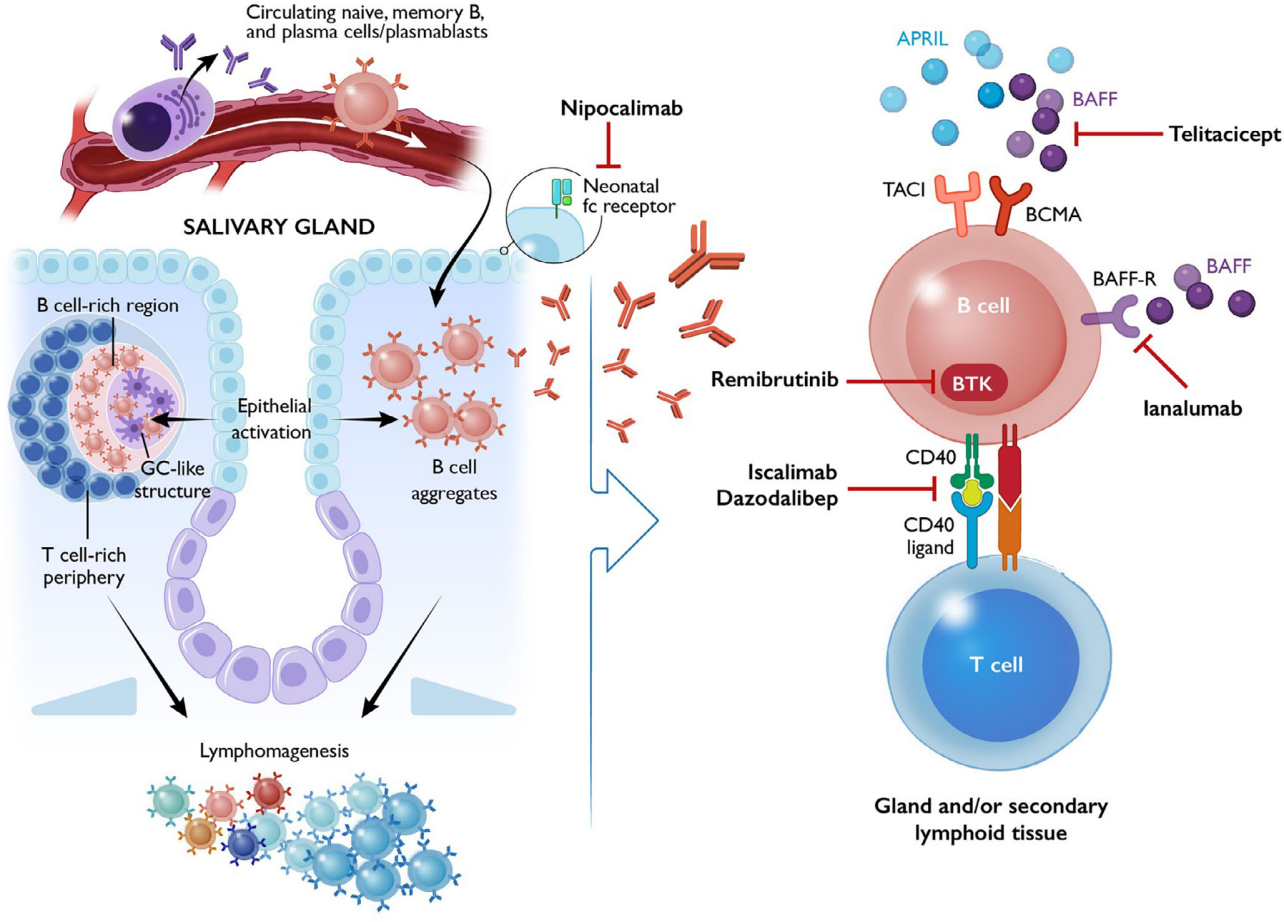
B cell pathways contributing to Sjögren disease (SjD) pathogenesis and drugs meeting phase II clinical trial endpoints targeting these pathways. B cells might migrate to local exocrine tissue, such as the salivary gland, and form B cell aggregates and germinal center (GC)‐like structures. These autoreactive B cells generate IgG autoantibodies that are hallmark features of SjD. Through chronic antigenic stimulation and driver mutations, among other processes, these B cells are capable of undergoing lymphomagenesis. Multiple drugs that target B cell–related pathways have met their phase II clinical trial endpoints and are shown targeting their respective pathways. Color figure can be viewed in the online issue, which is available at http://onlinelibrary.wiley.com/doi/10.1002/art.43404/abstract.

A 39‐year‐old woman presented with a 15‐year history of Sjögren disease (SjD) that was complicated by leukocytoclastic vasculitis and inflammatory arthritis. One year earlier, she had developed recurrent parotid swelling and required extensive dental work. She treated her parotid gland swelling with massage, warm compresses, and sugar‐free lozenges. She also had mild arthralgias of her metacarpophalangeal (MCP) and proximal interphalangeal (PIP) joints associated with one hour of morning stiffness. Her EULAR Sjögren's Syndrome Patient Reported Index (ESSPRI) score was 3. She had three pregnancies, with one complicated by congenital heart block. Her examination revealed diminished salivary pooling and a whole unstimulated salivary flow of 0.03 mL/5 min (SjD classification criteria^1^ normal >0.5 mL/5 min). She had normal Schirmer's testing (right: 17 mm/5 min, left: 35 mm/5 min; classification criteria normal >5 mm/5 min). Her bilateral parotid glands were enlarged (3 cm bilaterally) without enlargement of her submandibular glands. No cervical lymphadenopathy was appreciated on examination. She had no overt synovitis, but her bilateral index and long finger MCP and PIP joints of both hands were tender to palpation. Laboratory tests showed positive antinuclear, anti‐Ro/SSA, and anti‐La/SSB antibodies and rheumatoid factor (RF). Complete blood counts, metabolic panel, and anti‐Cyclic Citrullinated Peptide antibody were normal. Hand and foot x‐rays did not show any erosive changes. Salivary gland ultrasound showed hypoechoic and anechoic foci diffusely through the gland (Figure 1A). A core needle biopsy of the parotid gland showed lymphoepithelial lesions and germinal center (GC)‐like structures but no evidence of lymphoma (Figure 1, 2). Flow cytometry was negative for monoclonality. She was advised to start hydroxychloroquine for her inflammatory arthralgias and intermittent salivary gland swelling and to follow up in six months.

## Heterogeneity in SjD: endotyping as a tool to improve characterization and guide clinical care

SjD is highly heterogeneous. Although dryness, pain, and fatigue are hallmark features of SjD, a wide range of additional symptoms reflect the disease's impact on diverse organ systems. SjD causes a lymphocytic exocrinopathy, most notably of the lacrimal and salivary glands, resulting in profound dryness of the eyes and mouth. Beyond these hallmark symptoms, the individual patient experience in SjD varies widely, ranging from minimal dry eyes and dry mouth to life‐altering fatigue, cognitive difficulties, and a myriad of other symptoms. Beyond the lacrimal and salivary glands, SjD can affect multiple organ systems. A total of 30% to 50% of patients with SjD have extraglandular manifestations, including hematologic, pulmonary, renal, and neurologic involvement, among others. The variability of symptoms and organs impacted in patients with SjD results in several limitations to understanding and treating the disease. First, the heterogeneous presentation might reflect divergent pathogenic processes. As such, grouping phenotypically similar patients might provide improved insight into what drives these disparate presentations. Second, the variable presentations and natural history of SjD make it difficult to develop a treatment plan.^2^ Although most immunologic serology abnormalities predate SjD and these abnormalities tend not to change over time,^3^ the natural history of organ involvement is highly variable. For example, some patients with SjD present with dryness, and others present with organ‐threatening illness like interstitial lung disease, nephritis, neuropathy, or lymphoma. Each of these manifestations or combinations of manifestations might require a different approach to therapy. If a specific combination of clinical characteristics is well defined, then the disease course and treatment plan may become more predictable. Clear expectations regarding the disease's natural history and management will enhance clinicians’ understanding and enable them to communicate more effectively with patients. Thus, the improved characterization of SjD subtypes might lead to better clinician–patient relationships. A similar paradigm exists in systemic lupus erythematosus (SLE), another highly heterogeneous autoimmune disease.^4^

To address these limitations in studying and treating SjD, the research and clinical communities have moved to define endotypes. Endotypes are pathobiologically similar subgroups. Endotype generation in SjD is an evolving field of research,^5^ with approaches ranging from symptom based^6^, ^7^ to peripheral blood transcriptomics or proteomics^8^, ^9^ and glandular transcriptomics.^10^ Recently, Nguyen et al proposed a combined approach using symptoms, clinical disease activity, and laboratory studies to endotype patients with SjD.^11^ They identified three main endotypes of SjD: (1) a group with low systemic disease activity and high symptom burden (LSAHS), (2) a B cell active disease and low symptom burden (BALS) endotype, and (3) a high systemic activity (HSA) endotype. The HSA endotype had the highest disease activity burden, whereas the BALS endotype had the highest levels of immunoglobulins and RF, akin to the patient presented in our case. The LSAHS endotype had the highest symptom burden and lowest systemic disease activity and frequency of laboratory abnormalities. Each endotype displayed unique longitudinal features. The BALS endotype developed higher disease activity over time. In contrast, the HSA endotype demonstrated decreases in disease activity over time. Finally, there was no difference in the LSAHS endotype in either symptoms or signs over time. In addition to disease trajectory, we can glean prognosis from endotyping. Lymphoma exclusively occurred in the BALS and HSA groups. Thus, endotypes akin to those of Nguyen et al might help anticipate the disease course in SjD.

Though endotypes hold promise to improve patient care, this is an ongoing field of study. Future work includes longitudinal natural history studies to determine endotype evolution and prospective validation of endotypes in diverse independent cohorts. Furthermore, more prospective research is needed to determine if endotype classifications can be used to select effective therapeutics. Though endotype evaluation is an active field of research, greater understanding of this field is required before deploying these findings clinically.

## B cells as critical contributors to SjD pathogenesis

B cells play a crucial role in SjD pathogenesis by producing autoantibodies, presenting self‐antigens to autoreactive T cells, and releasing cytokines (Figure 2).^12^ The identification of genetic risk loci associated with SjD further underscores B cells as central mediators of disease. For example, genome‐wide association studies have identified polymorphisms in key B cell–related genes, including PR/SET domain 1, tumor necrosis factor alpha–induced protein 3 (TNFAIP3), B lymphoid tyrosine kinase, and C‐X‐C motif chemokine receptor 5 (CXCR5), that are associated with SjD.^13^

Beyond genetic predisposition, patients with SjD display distinct alterations in B cell subsets and tissue localization. Increased numbers of plasmablasts in the blood of patients with SjD correlate with elevated autoantibodies, higher disease activity, and lymphoma risk.^14^, ^15^ B cells, including activated B cells, are also found in the exocrine glands of patients with SjD.^12^ Interestingly, circulating memory B cells are decreased in patients with SjD compared to healthy individuals but are elevated in the salivary glands, suggesting that memory B cells may migrate to target tissues.^14^, ^15^, ^16^, ^17^, ^18^, ^19^ Antibody‐forming cells, including fully differentiated plasma cells, are also increased in the glands of patients with SjD.^14^, ^20^ Approximately 10% to 30% of patients with SjD have GC‐like structures in the salivary glands.^21^ Bonafide GCs in these glands express activation‐induced cytidine deaminase and CXCR5, whereas B cell aggregates do not.^22^ However, both GCs and B cell aggregates contain autoreactive B cells.^22^ An increased ratio of IgG‐/IgA‐producing B cells within the glands is associated with SjD and correlates with greater lymphocytic infiltration, as measured by the focus score.^23^, ^24^ More recently, “atypical memory B cells” in humans and “age‐associated B cells” in mice—proinflammatory B cell subsets linked to systemic autoimmunity and infections—have been identified in patients with SjD and mouse models.^25^, ^26^, ^27^, ^28^, ^29^, ^30^ The role of these inflammatory B cell populations in driving SjD pathogenesis remains an active area of investigation. The implementation of single‐cell multiomic technologies, such as single‐cell RNA sequencing and spatial transcriptomics, will enable investigators to better characterize B cell infiltration, spatial organization, and interactions within target organs affected by SjD.

Dysregulated B cell signaling and survival pathways contribute to SjD pathogenesis, highlighting key therapeutic targets under investigation. For example, BTK, a critical mediator of B cell receptor signaling, is elevated in B cells from patients with SjD.^31^ Additionally, levels of BAFF—a cytokine essential for B cell maturation, proliferation, and survival—are increased in both the serum and salivary glands of patients with SjD.^32^, ^33^, ^34^, ^35^ BAFF transgenic mice, who overproduce BAFF, develop systemic autoimmunity, including sialadenitis, and have an increased risk of lymphoma.^32^, ^36^ BAFF is not only produced by the myeloid compartment but also secreted by salivary gland epithelial cells, suggesting that the glandular epithelium is not a passive bystander in SjD pathogenesis but an active contributor to B cell activation and disease progression.^32^, ^34^, ^35^, ^37^ Together, these findings highlight B cells as key drivers of SjD pathogenesis and provide a strong rationale for targeting BAFF, BAFF receptor, and BTK in ongoing phase II/III clinical trials.

## When B cells go rogue: pathogenesis, risk factors, and clinical considerations in Sjögren lymphomagenesis

Non‐Hodgkin lymphoma is one of the most serious complications of SjD, affecting 5% to 10% of patients and significantly contributing to increased mortality rates.^38^, ^39^, ^40^, ^41^ Individuals with SjD have a 5 to >20 times higher risk of developing lymphoma compared to the general population, the highest among all autoimmune diseases—a wide range likely reflecting the heterogeneity of the populations studied.^38^ Notably, 98% of SjD‐associated lymphomas originate from B cells, further underscoring the critical role of B cells in SjD pathogenesis.^42^ The majority (>90%) of hematologic malignancies in SjD are mucosa‐associated lymphoid tissue (MALT) lymphoma and diffuse large B cell lymphoma.^42^, ^43^ Among these, low‐grade MALT lymphoma is the most common, with the salivary glands being the primary extranodal site (70%), though it can also arise in other locations, including the lungs, stomach, and ocular adnexal structures.^42^, ^43^

A leading hypothesis for lymphomatous transformation in SjD is that chronic antigen stimulation drives polyclonal B cell expansion, whereas the acquisition of driver mutations promotes monoclonal B cell proliferation. Intriguingly, using single‐cell DNA and RNA sequencing, Singh et al demonstrated that in SjD‐associated cryoglobulinemic vasculitis, RF B cells acquire lymphoma driver mutations before developing pathogenic V(D)J mutations that transform benign RF into pathogenic RF.^44^ This suggests that lymphoma driver mutations enable autoreactive pathogenic B cells to evade tolerance checkpoints, potentially explaining the link between cryoglobulinemic vasculitis and lymphoma development in patients with SjD.^44^ Additionally, both germline and somatic mutations in *TNFAIP3*, a key regulator of B cell activation, have been implicated not only in multiple autoimmune diseases but also in the increased lymphoma risk observed in SjD.^13^, ^45^, ^46^ Functional abnormalities in A20, the protein encoded by *TNFAIP3*, were detected in 77% SjD patients with MALT lymphoma and 29% of those with other histologic subtypes of lymphoma, further supporting its role in disease pathogenesis.^45^

Although these genetic and molecular insights highlight key mechanisms underlying lymphomagenesis in SjD, multiple clinical studies have also identified predictive factors that may help stratify patients at higher risk for lymphoma development. Several models identified factors or combinations of factors that are most predictive of lymphoproliferative disease. The first used a cohort of 723 patients with SjD, 38 of whom developed lymphoproliferative disease.^47^ Identified predictors of lymphoproliferative disease included parotid enlargement, palpable purpura, and low C4 levels at baseline.^47^ In a second cohort of 536 patients with SjD, 40 patients developed lymphoproliferative disease.^48^ In this cohort, neutropenia, cryoglobulinemia, splenomegaly, lymphadenopathy, and low C4 levels predicted non‐Hodgkin lymphoma.^48^ In a third cohort of 661 patients with SjD, 40 patients developed non‐Hodgkin lymphoma^49^; the investigators identified low C4, cryoglobulins, anti‐La/SSB antibodies, and leukopenia as predictors of lymphoproliferative disease. Finally, a fourth cohort of 381 patients with SjD, 92 of whom had non‐Hodgkin lymphoma, identified salivary gland enlargement, lymphadenopathy, Raynaud phenomenon, anti‐Ro/SSA and/or anti‐La/SSB autoantibodies, RF, monoclonal gammopathy, and low C4 as predictors for non‐Hodgkin lymphoma development.^50^ Beyond these models, high focus score and GCs on labial salivary gland biopsies also predict the risk of lymphomagenesis.^51^, ^52^ High disease activity, excluding lymphoma, also is associated with increased risk of lymphoma.^53^ Furthermore, imaging such as salivary gland ultrasound and computed tomography (CT)/positron emission tomography (PET) might also help predict glandular lymphoma risk,^54^, ^55^ particularly in the setting of a parotid PET‐CT standardized uptake value maximum ≥4.7.^56^ For example, parotid lymphomas seem to occur in salivary glands with hypoechoic or anechoic lesions (grade 2 or 3 using the Outcome Measures in Rheumatology [OMERACT] salivary gland ultrasound scoring system).^55^, ^57^ Our patient had salivary gland enlargement, high disease activity, positive anti‐Ro/SSA and La/SSB antibodies, positive RF, and a high OMERACT salivary gland ultrasound score. These features indicate that she might be at higher risk for developing lymphoma.

Lymphoma suspicion should be rooted in the history and physical examination of our patients and supplemented with supportive laboratory and imaging findings. MALT lymphoma of the gland is the most common lymphoproliferative disease in SjD and characteristically presents with recurrent or persistent swelling of salivary glands. Patients who have B symptoms, lymphadenopathy, or recurrent parotitis with a laboratory profile suggestive of higher risk for lymphomatous transformation warrant an evaluation. Salivary gland ultrasound or CT/PET can help identify ideal biopsy sites. Ultimately, treatment decisions should be made collaboratively between the patient and hematology/oncology and rheumatology providers.

## Advancing SjD treatment: B cell–targeted therapies and the evolving therapeutic landscape

### Successful phase II trials: a new era in SjD treatment

As discussed above, B cells play a central role in SjD pathogenesis, even more so when focused on B cell–enriched endotypes. Despite the mechanistic promise of B cell–targeted therapies, past randomized placebo‐controlled trials with the anti‐CD20 therapy, rituximab, did not achieve their primary endpoints.^58^, ^59^ It is hypothesized these early failures might have been related to the clinical trial design, including broad inclusion criteria and endpoints that were not sensitive to therapeutic intervention. Alternatively, rituximab might fail to deplete B cells in salivary gland tissue.^60^ In recent years, however, the SjD treatment landscape has shifted, and multiple putative drugs have achieved their primary endpoint in phase II trials, many of which target B cell–related pathways (Table 1). These early successes represent a potential paradigm shift in SjD, from symptomatic management to therapies that might treat or even prevent the progression of the disease. Some of these recent trial successes may be partly explained by the significant evolution of inclusion criteria and endpoints of SjD clinical trials over time. Most trials now require moderate to high systemic disease activity, as measured by the EULAR Sjögren's Syndrome Disease Activity Index (ESSDAI)^61^, ^62^ and positive anti‐SSA antibody, for entry.

**Table 1:** Successful SjD phase II randomized controlled trials targeting B cells*.

Drug	Target	Inclusion	Primary endpoint	Primary endpoint data	*P* value
Dazodalibep	CD40 ligand	SjD by ACR/EULAR Positive anti‐SSA antibody or positive RF	**–**	LSM ± SE	**–**
Cohort 1	–	ESSDAI ≥5	Change from baseline in ESSDAI	LSM ± SE Dazodalibep, −6.3 ± 0.6; placebo, −4.1 ± 0.6	0.0167
Cohort 2	–	ESSDAI <5 ESSPRI ≥5 >0.1 mL/min salivary flow	Change from baseline in ESSPRI	LSM ± SE Dazodalibep, −1.8 ± 0.2; placebo, −0.5 ± 0.2	0.0002
Ianalumab	BAFF receptor	SjD by AECG ESSDAI ≥6 ESSPRI ≥5 Anti‐SSA antibody positive >0.1 mL/min salivary flow	Change from baseline in ESSDAI and dose‐dependent response at week 24 of placebo‐subtracted ESSDAI change from baseline	Placebo‐adjusted LSM (95% CI) from baseline 300 mg ianalumab: −1.92 (−4.15 to −0.32)	Dose–response change in ESSDAI from baseline *P* < 0.025 in four models; *P* = 0.06 in one model
Iscalimab	CD40	SjD by ACR/EULAR Positive anti‐SSA antibody >0.1 mL/min salivary flow	–	Placebo‐adjusted LSM from baseline (95% CI)	Dose–response change in ESSDAI from baseline *P* = 0.0041 in one of four models
Cohort 1	–	ESSDAI ≥5 ESSPRI ≥5 Anti‐SSA antibody positive	Dose–response change from baseline in ESSDAI^a^ Cohort who took 150 mg Cohort who took 600 mg	Placebo‐adjusted LSM from baseline (95% CI) 150 mg: −3.0 (−4.9 to −1.1) 600 mg: −2.9 (−4.9 to −1.0)	–
Cohort 2	–	ESSDAI <5 ESSPRI ≥5 Impact of Dry Eye on Everyday Life score ≥ 30	Change from baseline in ESSPRI did not achieve its primary endpoint	Placebo‐adjusted LSM from baseline (95% CI), 600 mg: −0.57 (−1.3 to 0.15)	–
Nipocalimab	FcRN	SjD by ACR/EULAR clinESSDAI ≥6 Anti‐SSA antibody positive	Change from baseline in clinESSDAI	LSM change from baseline (90% CI): 5 mg/kg nipocalimab, −0.34 (−1.71 to 1.03) 15 mg/kg nipocalimab, −2.65 (−4.03 to −1.28)	5 mg/kg, *P* = 0.681 15 mg/kg, *P* = 0.002
Remibrutinib	BTK	SjD by ACR/EULAR ESSDAI ≥5 ESSPRI ≥5 Anti‐SSA antibody positive >0 mL/min salivary flow	Change from baseline in ESSDAI	LSM change from baseline (95% CI): −2.86 (−4.71 to −1.01)	0.003
Telitacicept	BAFF and APRIL	SjD by ACR/EULAR ESSDAI ≥5 Anti‐SSA antibody positive	Change from baseline in ESSDAI	Placebo‐adjusted LSM change from baseline (90% CI): 160 mg, −4.3 (−7.0 to −1.6) 240 mg, −2.7 (−5.6 to 0.1)	160 mg, *P* = 0.002 240 mg, *P* = 0.056

* ACR, American College of Rheumatology; AECG, American‐European Consensus Group; CI, confidence interval; clinESSDAI, Clinical EULAR Sjögren's Syndrome Disease Activity Index; ESSDAI, EULAR Sjögren's Syndrome Disease Activity Index; ESSPRI, EULAR Sjögren's Syndrome Patient Reported Index; FcRN, neonatal Fc receptor; LSM, least squares mean; RF, rheumatoid factor; SjD, Sjögren disease.

^a^ The cohort who took 300 mg did not meet its primary endpoint and is therefore not shown.

One such trial evaluated ianalumab, an anti‐BAFF receptor antibody that can deplete B cells, in patients with anti‐SSA positivity and SjD with moderate to high symptom burden and disease activity. It achieved its primary outcome of dose–response improvement in disease activity from baseline.^63^ Ianalumab appears to improve salivary flow but did not show clear benefit on SjD symptoms. In the extension study to 52 weeks, ianalumab seemed to maintain efficacy.^61^

The anti‐neonatal Fc receptor (FcRN) antibody nipocalimab reported promising phase II clinical trial results at the 2024 American College of Rheumatology (ACR) annual conference. Anti‐FcRN antibodies prevent IgG recycling, reducing circulating autoantibodies. Nipocalimab treatment of patients with SjD with moderate or severe disease activity and positive anti‐Ro/SSA antibodies resulted in improved disease activity. The resulting paper was recently published.^64^

Remibrutinib is a BTK inhibitor^65^ that was tested in a phase II study of patients with anti‐SSA positivity and SjD with moderate to high disease activity. BTK inhibitors reduce B cell proliferation and drive B cell apoptosis. Remibrutinib improved disease activity but did not significantly improve symptoms or objective measures of salivary flow.

Telitacicept is a humanized protein that binds and inhibits B lymphocyte stimulator (BLyS; ie, BAFF) and APRIL. Through inhibition of BLyS and APRIL, telitacicept inhibits differentiation, maturation, and survival of B cells. In a phase IIb study of patients with anti‐Ro/SSA antibody positivity and SjD with moderate to high disease activity, 160 mg telitacicept significantly improved ESSDAI compared to placebo; however, the 240‐mg dose did not improve ESSDAI significantly compared to placebo.^66^ Fatigue assessments, as measured through the multidimensional fatigue inventory, improved significantly in both doses.

In contrast to the aforementioned studies, the iscalimab and dazodalibep trials stratified patients by endotype. These drugs target the CD40–CD40 ligand interaction that drives GC formation, Ig class switching, and inflammatory cytokine production. Iscalimab is a humanized monoclonal antibody against CD40 with a modified Fc domain that makes its Fcγ‐dependent effects nonfunctional.^67^ Iscalimab was tested in two cohorts of patients with anti‐Ro/SSA positivity and SjD: a cohort with high disease activity or a cohort with high symptom burden who also had significant dryness or fatigue burden.^68^ The cohort with high disease activity achieved its primary endpoint of dose–response improvement of disease activity. The cohort with high symptom burden did not meet its primary endpoint of symptom improvement but did show significant improvement in the ESSPRI dryness domain. Dazodalibep is an anti‐CD40 ligand antagonist^69^ that was uniquely also tested in two populations with anti‐Ro/SSA positivity or RF positivity: (1) a cohort with high disease activity, and (2) a cohort with low disease activity and high symptom burden. Dazodalibep achieved its primary endpoint for each respective cohort. It improved disease activity among patients with high disease activity and it improved symptom burden in patients with low disease activity and high symptom burden. Salivary flow did not improve significantly in either group. These successful phase II studies offer a promising glimpse into a growing therapeutic landscape, highlighting the potential for transformative advances in SjD treatment.

### Novel emerging therapeutic strategies in SjD

Chimeric antigen receptor (CAR) technology has revolutionized targeted immunotherapy, offering new avenues for treating both malignancies and autoimmune diseases.^70^ CARs are genetically engineered transmembrane receptors inserted into immune cells, like T cells.^70^ They typically consist of an antigen‐binding domain, hinge, transmembrane, and intracellular signaling domains.^70^ CAR‐T cells, which can be autologous or allogeneic, have shown remarkable efficacy in treating cancers, particularly hematologic malignancies, by specifically binding to and lysing oncogenic cells.^70^ More recently, CAR technology has been applied to the treatment of systemic autoimmune diseases such as SLE.^71^, ^72^, ^73^, ^74^, ^75^, ^76^, ^77^, ^78^, ^79^ CD19‐targeting CAR‐T cells, which deplete B cells, have shown promising efficacy in lupus nephritis.^71^, ^72^ Similarly, BCMA CAR‐T cells, designed to eliminate plasma cells, are used in multiple myeloma.^70^ Given the central roles of B cells and plasma cells in SjD, both CD19 and BCMA CAR‐T cell therapies warrant further investigation as potential treatments. Beyond depleting pathogenic cells, CAR technology is also being explored to develop regulatory cell products that suppress autoreactive cells, presenting a novel strategy for treating autoimmune diseases.

Monoclonal antibodies that recognize single antigens, like a cytokine or cytokine receptor, have been used for the treatment of rheumatic diseases for over 20 years and have revolutionized patient care. This advancement has paved the way for more sophisticated antibody‐based therapies, such as bispecific T cell engagers (BiTEs). BiTEs are bispecific antibodies that recognize an antigen on a target cell and endogenous T cells to direct T cells toward target cells for precise T cell–mediated destruction.^80^, ^81^ Blinatumomab (CD3/CD19) and teclistamab (CD3/BCMA) are BiTEs that target B cells and plasma cells, respectively.^80^, ^81^ Akin to CAR‐T cells, BiTEs were initially developed for the treatment of hematologic malignancies, but there is emerging evidence for efficacy in autoimmune diseases. For example, blinatumomab has been used in six patients with rheumatoid arthritis (RA), and teclistamab has been used in five patients with systemic autoimmunity (RA, myositis, SjD, systemic sclerosis, and SLE), with disease improvement in all patients.^82^, ^83^, ^84^ Bispecific and BiTE antibody engineering offers a flexible platform for the treatment of systemic autoimmune diseases such as SjD. B cell–targeted CAR‐T, bispecific, and BiTE therapies have a more potent and sustained effect on B cell depletion than CD20‐targeted monoclonal antibodies like rituximab. These agents target plasmablasts, early plasma cells, and memory B cell populations,^85^ both in the periphery and end organs.

## Conclusions

The patient returned nine years later. Since her last visit with a rheumatologist, she had been observed regularly by a ears, nose, and throat specialist for recurrent left parotid swelling. Her ultrasound showed diffuse anechoic and hypoechoic foci comprising most of the gland tissue and now showed one dominant cystic lesion. This cystic lesion was very hypoechoic/anechoic, was oval, and had well‐defined margins without septa or posterior acoustic enhancement (Figure 1F). She underwent drainage of a left parotid cyst every six months until she was ultimately referred for a parotidectomy. Pathology revealed MALT lymphoma (Figure 1G–J).

This patient presented with an endotype most consistent with a B cell active phenotype, which is associated with a higher risk of lymphoma. She had several clinical risk factors for lymphoma development in her history and laboratory tests. She had recurrent parotid swelling, a history of leukocytoclastic vasculitis, and her serologies were notable for a high‐titer RF, all of which have been linked to an increased likelihood of lymphoma. Her initial parotid biopsy also demonstrated GCs, indicative of chronic B cell activation and a heightened risk of lymphomatous transformation.

This case underscores the critical need for vigilant surveillance in patients with SjD with known risk factors because early detection of lymphomas can be crucial for timely intervention and optimal outcomes. Such considerations are particularly important given the complex relationship between SjD and lymphoma, which may have implications for both oncologic and autoimmune disease management. Although the treatment of SjD‐associated lymphoma is an area of controversy, actively under investigation and largely under the purview of hematology, there are data that indicate treatment of SjD‐associated lymphoma might also improve disease activity in SjD.^86^

Despite being nearly as common as RA, SjD still lacks US Food and Drug Administration (FDA)‐approved disease‐modifying therapies. SjD heterogeneity is a major contributor to the lack of FDA‐approved disease‐modifying therapies. Endotyping is an approach that is closing this gap, creating pathobiologically similar subtypes to reduce heterogeneity and identifying B cell–driven disease endotypes. Patients with these B cell endotypes seem to have greater risks for lymphoma and are one of the populations that might benefit from the aforementioned burgeoning therapies. The therapeutic landscape in SjD is rapidly expanding, ushering in a new era of innovation and long‐overdue breakthroughs for this overlooked disease.

## AUTHOR CONTRIBUTIONS

All authors contributed to at least one of the following manuscript preparation roles: conceptualization AND/OR methodology, software, investigation, formal analysis, data curation, visualization, and validation AND drafting or reviewing/editing the final draft. As corresponding author, Dr McCoy confirms that all authors have provided the final approval of the version to be published, and takes responsibility for the affirmations regarding article submission (eg, not under consideration by another journal), the integrity of the data presented, and the statements regarding compliance with institutional review board/Declaration of Helsinki requirements.

## Supporting information


**Disclosure Form**:
